# Simulation of ITD-Dependent Single-Neuron Responses Under Electrical Stimulation and with Amplitude-Modulated Acoustic Stimuli

**DOI:** 10.1007/s10162-021-00823-1

**Published:** 2022-03-25

**Authors:** Hongmei Hu, Jonas Klug, Mathias Dietz

**Affiliations:** Department of Medical Physics and Acoustics and Cluster of Excellence “Hearing4all”, University of Oldenburg, 26129 Oldenburg, Germany

**Keywords:** Interaural time differences, Bilateral cochlear implant, Binaural modeling, LSO, Excitatory-inhibitory interaction, Rate limitation

## Abstract

Interaural time difference (ITD) sensitivity with cochlear implant stimulation is remarkably similar to envelope ITD sensitivity using conventional acoustic stimulation. This holds true for human perception, as well as for neural response rates recorded in the inferior colliculus of several mammalian species. We hypothesize that robust excitatory-inhibitory (EI) interaction is the dominant mechanism. Therefore, we connected the same single EI-model neuron to either a model of the normal acoustic auditory periphery or to a model of the electrically stimulated auditory nerve. The model captured most features of the experimentally obtained response properties with electric stimulation, such as the shape of rate-ITD functions, the dependence on stimulation level, and the pulse rate or modulation-frequency dependence. Rate-ITD functions with high-rate, amplitude-modulated electric stimuli were very similar to their acoustic counterparts. Responses obtained with unmodulated electric pulse trains most resembled acoustic filtered clicks. The fairly rapid decline of ITD sensitivity at rates above 300 pulses or cycles per second is correctly simulated by the 3.1-ms time constant of the inhibitory post-synaptic conductance. As the model accounts for these basic properties, it is expected to help in understanding and quantifying the binaural hearing abilities with electric stimulation when integrated in bigger simulation frameworks.

## INTRODUCTION

Bilateral cochlear implant (CI) users are able to coarsely localize sound sources. They are better at this than unilateral CI users, but at the same time much worse than normal-hearing (NH) listeners. With their clinical devices, they can exploit interaural level differences (ILDs), while obtaining little to no benefit from interaural time differences (ITDs) (Seeber and Fastl 2008). However, when stimulating a single left and a single right CI electrode at a low rate, almost all bilateral CI users have demonstrated some degree of ITD sensitivity (for review, Kan and Litovsky 2015; Laback et al. 2015). The median detection thresholds are 144 µs, 12 times larger than those of young and well-trained NH listeners (Laback et al. 2015). In addition, ITD sensitivity is limited to stimulation rates below 300–500 pulses per second (pps) (Ihlefeld et al. 2015; Laback et al. 2007; van Hoesel 2007), in contrast to the NH limit near 1400 Hz for pure tones (Brughera et al. 2013). This moderate level of ITD sensitivity is very similar to the envelope ITD sensitivity of NH listeners (Dietz 2016).

At the level of the auditory nerve (AN), the trend is opposite: phase locking to electric stimulation is generally better than with acoustic stimulation (Dynes and Delgutte 1992; Hartmann et al. 1984; Shepherd and Javel 1997). Some AN fibers can phase lock to electrical pulse trains at rates of 5000 pps (e.g., Miller et al. 2008) or even above 5000 pps (e.g., Dynes and Delgutte 1992). At the level of the inferior colliculus (IC), the picture is less consistent. Some studies report ITD sensitivity with electric stimulation similar to acoustic stimulation (Rosskothen-Kuhl et al. 2021; Vollmer 2018), some report a slightly lower rate limit and less robust tuning (Smith and Delgutte 2007), and some report a low rate limit similar to the perceptual limit (Chung et al. 2016; Hancock et al. 2013). It can be inferred that the temporal information present at the level of the AN cannot be exploited by the brain to the same degree, but experimental challenges have so far prevented deeper insights. Moreover, significant differences between related studies at each stage impose additional obstacles on theory development. A variety of factors, such as species differences, deafening procedure, duration of deafness, implantation, anesthetics, stimulation procedure, neuron search techniques, and selection criteria, and an equally long list of differences in the human psychoacoustic experiments, severely limit across-study comparisons.

Computational simulation of the whole process can improve comparability and deepen our understanding of the functional relations. Specifically, it can quantify the influence of some procedural differences, and it can be instrumental in developing and consolidating hypotheses about the partial loss of temporal information. Here, we propose a simulation framework for both acoustic and electrical stimulations of binaurally sensitive model neurons. It is used to quantify the influence of stimulus types and parameters, and for comparing between acoustic and electrical stimulations.

Previous studies on this topic simulated excitatory-excitatory neurons (e.g., Chung et al. 2015; Colburn et al. 2009). They were able to reproduce peak-type rate-ITD functions (from e.g., Smith and Delgutte 2008) even at high rates such as 1000 pps. However, most human bilateral CI users cannot exploit ITDs at these rates and rather show an ITD sensitivity similar to NH envelope ITD sensitivity, e.g., a limit around 300–500 pulses or cycles per second. Envelope ITD sensitivity is commonly associated with LSO processing (Tollin 2003), which is why Dietz (2016) hypothesized that the LSO pathway may be crucial for bilateral CI users. Therefore, a goal of the present study is to complement the existing functional analysis of EE-type models with LSO-type EI processing and to discuss if this pathway has potential for future studies that model behavioral ITD sensitivity in bilateral CI users.

The general philosophy of the present study was to use “off the shelf” model components with default parameters, rather than fitting parameters to isolated neurons or data sets. The focus was on three main factors: stimulation level, stimulation rate, and stimulation type. The latter describes different amplitude-modulation shapes presented acoustically, unmodulated pulse trains presented electrically, and high-rate pulse trains with sinusoidal amplitude modulation presented electrically. Additionally, as an application example, we varied the inhibitory post-synaptic conduction time constant to demonstrate its influence on the ITD rate limit.

## METHODS AND MATERIALS

### Modeling

In order to compare the model outputs with acoustic stimulation and with electrical stimulation, the model framework of Klug et al. (2020) was adopted in this study. In a nutshell, it is an EI-model neuron receiving bilateral input from either an acoustic or an electrical model of the auditory periphery. The model code and data for reproducing the results are freely available on Zenodo (Hu et al. 2021).

#### Model of the Acoustically Stimulated Auditory Nerve

The periphery model of Bruce et al. (2018) was applied in the same fashion as in Klug et al. (2020) for simulations of acoustic hearing. It transforms the acoustic stimuli into spiking patterns of medium-spontaneous-rate (MSR, representative spontaneous rate is 0.5–18 spikes per second) AN fibers arrayed along the tonotopic axis. The input to the model is a stimulus in the form of a pressure waveform. The output of the model is given by a spike generator that produces a series of AN spikes. All simulated AN fibers had a characteristic frequency (CF) equal to the stimulus carrier frequency (CF = 8000 Hz unless otherwise stated). The model parameters were kept unchanged from (Bruce et al. 2018; Zilany et al. 2014, 2009).

#### Model of the Electrically Stimulated Auditory Nerve

To simulate electrical hearing, the auditory periphery model was substituted by the AN model of Hamacher (2004) in the implementation of Fredelake and Hohmann (2012). This model consists of four stages: cell membrane, membrane noise, refractory period, and latency and jitter. The response probability of the models only depends on two parameters: (1) The membrane voltage after the first pulse phase. It is calculated as a deterministic, leaky, integrate-and-fire model (Gerstner and Kistler 2002) extended with a zero-mean Gaussian noise source (membrane noise) to simulate stochastic behavior of the AN fibers (Hamacher 2004) influenced by the threshold potential, stimulation current, and the first-phase duration. (2) The time difference between the onset of the current pulse and the last action potential. The effective firing time depends on the stimulation time, the discharge time of the membrane capacitance when an action potential is generated, the latency, and the jitter. The mean and standard deviation of the latency and jitter were derived from the data of Miller et al. (1999a; 1999b). All parameters were the same as in (Fredelake and Hohmann 2012; Hamacher 2004). It should be noted that the AN model doesn’t include adaptation and assumes the same threshold level for all AN fiber inputs, which is unlikely to be the case in a real system. Tonotopic considerations and spread of excitation are irrelevant for the purpose of this study. In other words, the 1D model is independent of the location of the stimulated CI-electrode along the basilar membrane and only those AN fibers distributed at the corresponding position were analyzed and passed onto the binaural interaction stage.

#### Binaural Neuron Model

**Fig. 1:**
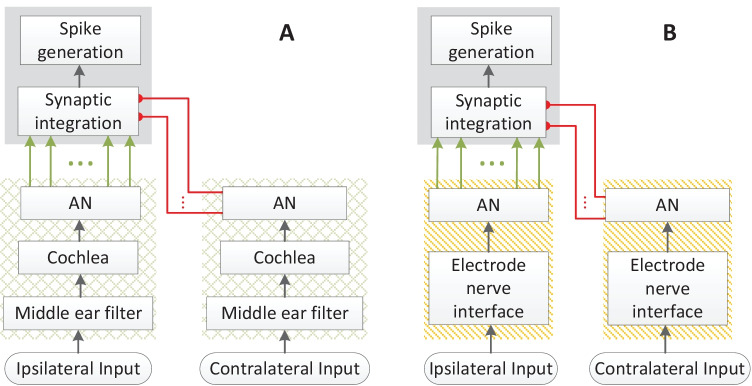
Structures of acoustic- **A** and electric- **B** model diagrams. Both model structures constitute the periphery and the excitatory-inhibitory (EI) integration stages. Both acoustic periphery (within green-crosshatch boxes) and electric periphery (within orange-striped boxes) receive the binaural stimulus as the inputs, and the excitatory-inhibitory (EI) integration stage (within the gray boxes) that bilaterally receives the excitatory (arrow) and inhibitory (bullet) outputs of the periphery

At the binaural interaction stage, a single EI-model neuron as in Klug et al. (2020), modified from the coincidence-counting LSO model of Ashida et al. (2016), was used. Unless otherwise stated, the default EI-model parameters of Klug et al. (2020) were used. Figure 1 shows the structures of acoustic- (Fig. 1(A)) and electric- (Fig. 1(B)) model diagrams. Briefly, it receives excitatory synaptic inputs from ipsilateral AN fibers and inhibitory inputs from contralateral AN fibers (see Klug et al. 2020 for a more detailed description of the model). In this study, for a specific CF or CI electrode, 20 MSR AN fibers were simulated on the ipsilateral ear to generate excitatory input and 8 on the contralateral ear for generating the inhibitory input to the EI-model. The length of the rectangular excitatory coincidence window and the rectangular inhibitory window (*W*_inh_) was 1.1 ms and 3.1 ms, respectively. The response threshold was 3, the inhibitory gain for increasing the weight of inhibitory inputs was 2, and the length of the refractory period was 1.6 ms.

### Stimuli

#### Acoustic Stimuli

To coarsely replicate the AN excitation patterns from CI stimulation in NH listeners, a pulse-mimicking envelope is often multiplied on a high-frequency carrier (Goupell et al. 2013). The AN can safely be assumed to phase-lock only to the pulse-mimicking envelope, generating comparable temporal information at least at the level of the AN and at pulse rates below the auditory-filter bandwidth. For example, past studies have employed Gaussian envelope (GE) tones (Bernstein et al. 2018; Ehlers et al. 2016; Goupell et al. 2013) or filtered clicks (e.g., Baumgärtel et al. 2017; Hu et al. 2017; Majdak and Laback 2009). Such stimuli have a wider spectrum than the sinusoidally amplitude-modulated (SAM) tones and the transposed tones (Goupell et al. 2013) commonly used in NH studies (e.g., Bernstein and Trahiotis 2002, 2003; Blanks et al. 2008; Ehlers et al. 2016; Monaghan et al. 2015). Among these studies, ITD sensitivity has been compared in Goupell et al. (2013) using GE tone pulses and non-Gaussian-shaped pulses, and in Ehlers et al. (2016) using GE tones and transposed tones in the same subjects. However, no study systematically tested more than two stimulus types on the same subjects, and it is still unclear whether ITD sensitivity differs for different CI simulating stimuli and which one may be most similar to actual electrical pulse trains. Thus, in the current study, four types of acoustic stimuli were selected. The duration of each stimulus was set to 1 s, with a 10-ms sin^2^ gating. In order to obtain the envelope ITD (ITD_ENV_) tuning curves for different modulation frequencies (*f*_*m*_), a range of ITD within [− 4, 4] ms or IPD within one period ([$-\pi,\pi$]), with a step size of 0.1 ms, 0.2 ms, or 0.05$\pi$, was tested at different stimulation levels. Positive ITDs represent contralateral-leading. Consistent with Klug et al. (2020), the ITDs were inserted on the acoustic AN spike trains to reduce computational demand, and responses within the first 200 ms were discarded.

#### SAM Tones

As one of the most commonly used stimulus classes, high-frequency SAM tones were generated digitally as in Bernstein and Trahiotis (2012) according to Eq. (1):

1 $$s(t) = a \mathrm{sin}\left(2\pi \it {{f}_{c} t}\right)\left(1-\mathrm{cos}2 \pi \it{{f}_{m}t}\right)$$

**Fig. 2:**
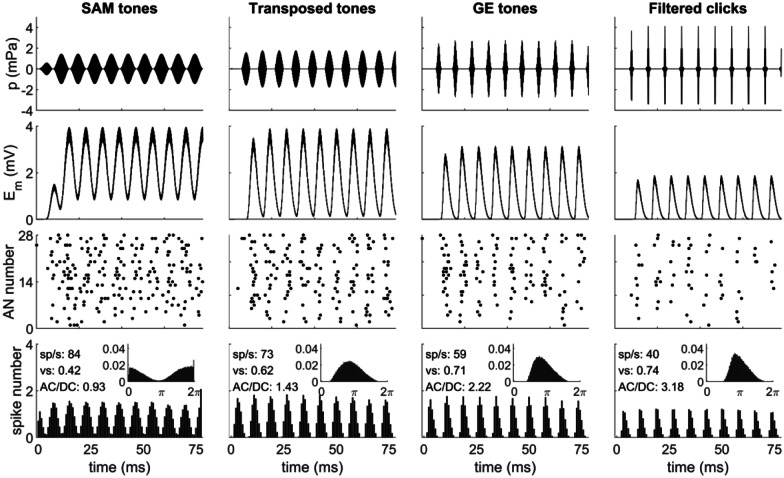
Waveforms of example acoustic stimuli (modulation frequency *f*_*m*_ = 128 Hz, carrier frequency *f*_*c*_ = 4000 Hz, stimulus level is 30 dB SPL) and the corresponding responses of the acoustic periphery models (CF = 4000 Hz) to SAM tones (column 1), transposed tones (column 2), GE tones (column 3), and filtered clicks (column 4) within [0 78] ms: row 1, stimulus-sound waveform; row 2, the IHC receptor potential; row 3, the spike raster plot of 28 AN fibers in one repetition; row 4, the average PSTH and the period histogram (small inset) of 50 repetitions

Unless otherwise stated, the default test carrier frequency was *f*_*c *_= 8000 Hz. Different modulation frequencies *f*_*m*_ were tested. Figure 2 shows an example of an SAM tone (top row column 1).

#### Transposed Tones

Transposed stimuli were designed by van de Par and Kohlrausch (1997) to better mimic low-frequency tonal responses in high-frequency AN fibers. To simulate the functional role of hair cells, a low-frequency base stimulus is half-wave rectified and subsequently low-pass filtered. The output serves as the modulator and is multiplied on a high-frequency carrier. Transposed tones clearly have sharper envelopes than SAM tones and have been extensively used in binaural studies with NH adult listeners (Bernstein and Trahiotis 2002, 2003). Here, the transposed tones were generated as in (Bernstein and Trahiotis 2002), and the default carrier frequency was *f*_*c*_ = 8000 Hz, with various modulation frequencies. Figure 2 shows one example of a transposed tone (top row column 2).

#### Gaussian Envelope Tones

In this study, the band-limited, constant-amplitude Gaussian envelope (GE) tone pulse trains were generated as described in (Bernstein et al. 2018; Goupell et al. 2013). Briefly, they were generated in the time domain by applying a Gaussian-shaped envelope to a tonal carrier at the desired stimulation pulse rate (in pps). Here, GE tones were presented at different pulse rates (which correspond to the *f*_*m*_ in SAM and transposed tones) and different levels, with the duration of the individual pulses manipulated to generate an equivalent rectangular bandwidth of 1.5 mm as defined by Greenwood (1990). Figure 2 (top row column 3) shows an example of a GE tone train with an equivalent rectangular bandwidth of 1691 Hz and an equivalent rectangular pulse duration of 0.6 ms.

#### Filtered Click Trains

Bandpass-filtered click trains, similar to (McKay and Carlyon 1999), make up another class of commonly-used stimuli for simulating CI pulsatile stimulation. When investigating temporal effects in NH listeners, it provides a good approximation of the electrical stimulation in CIs (Carlyon and Deeks 2013; Laback et al. 2007; Majdak and Laback 2009; Majdak et al. 2006; McKay and Carlyon 1999). Although the response of an auditory filter to bandpass-filtered click trains has a clearly reduced envelope sharpness when compared to electrical pulse trains, the resulting envelope is still sharper than for SAM or transposed tones. In this study, unmodulated bandpass-filtered click trains were created similar to (Hu et al. 2017): firstly, rectangular condensation pulses of fixed width (10 µs) were generated at different pulse rates; secondly, band-pass filtering was performed using a 2nd-order constant *Q* = 0.5 Butterworth filter. Figure 2 (top row column 4) shows an example of a bandpass-filtered click train. Depending on the context, their click rate is also referred to as either pulse rate or modulation frequency.

#### Electric Stimuli

One of the most commonly used stimuli for ITD sensitivity in both human CI users and in electrically stimulated animals is unmodulated low-rate pulse trains (e.g., Chung et al. 2016; Smith and Delgutte 2007). However, CI processors typically encode the sound envelope in each frequency band by amplitude-modulating (AM) high-rate pulse trains. Smith and Delgutte (2008) investigated the ITD sensitivity of IC neurons using SAM high-rate pulse trains (carrier rate of 1000 or 5000 pps). They found that many IC cells were sensitive to ITD in both the envelope (ITD_ENV_) and the pulse carrier for appropriate modulation frequencies and carrier rates; ITD_ENV_ tuning generally improved with increasing modulation frequency up to the maximum tested (≤ 160 Hz) that elicited a sustained response in a neuron; ITD sensitivity to carrier pulses was present in about half the neurons for 1000-pps carriers and was nonexistent at 5000 pps. High-rate stimulation above the AN phase-locking limit has been proposed to improve a CI user’s speech perception (Rubinstein et al. 1999). It has been demonstrated that CI users can exploit ITDs in these envelopes, while being insensitive to the timing of the high-rate carrier pulses (Wilson and Dorman 2008).

In the current study, both unmodulated, low-rate pulse trains (mostly < 1000 pps) and SAM, high-rate pulse trains (1000 pps and 5000 pps) were systematically tested with the electrical model. The focus was on reproducing the stimulus conditions from Chung et al. (2016) and Smith et al. (2007; 2008), but, in the absence of experimental constraints, some additional conditions were simulated.

The unmodulated and the SAM high-rate electrical stimuli were constant-amplitude and amplitude-modulated biphasic pulse trains, respectively (cathodic/anodic, 100-µs phase duration; although the CI AN model is indifferent to polarity). The envelope of the SAM pulse-trains was $s\left(t\right)= [1-\mathrm{cos}\left(2\pi \it{{f}_{m}t}\right)]/2$, with a fixed carrier rate (1000 pps or 5000 pps). In contrast to Smith and Delgutte (2008), static ITDs, instead of slowly changing ITDs, were applied in all simulations. The pulse rate (unmodulated pulses) or *f*_*m*_ (SAM high-rate pulse trains for a fixed carrier rate) was varied parametrically. The upper limit of *f*_*m*_ was up to 40% of the respective pulse rate, which is quite high, and means that the actual envelope is not well reconstructed by interpolating the pulses in these conditions. As in the acoustic simulation, an [− 4, 4] ms ITD range or an IPD within one period ([$-\pi,\pi$]) was tested at different stimulation levels compared to a reference threshold. The duration of each electrical stimulus was 600 ms, with a 10 ms sin^2^ gating.

Motivated by Smith and Delgutte (2007, 2008), a 40-pps unmodulated pulse train was used as the “standard” stimulus to obtain a reference threshold of unmodulated pulse trains, and a 40-Hz sinusoidally amplitude-modulated 1000-pps pulse train served as a reference for all SAM pulse trains. More specifically, a reference threshold level was defined as the current of the “standard” stimulus when ipsilateral-only stimulation evoked at least one spike/s (sp/s) in the EI-model neuron. For the 5000 pps SAM, the same threshold current was used as for the 1000 pps condition, as in Smith and Delgutte (2008). For the electrical stimulation, the presentation level is stated in dB re threshold (dB thr), which is 240 µA for all unmodulated, and 130 µA for all SAM pulse trains. The latter is lower due to the higher pulse rate. It should be noted that the unit s^−1^ was introduced in the results for simplicity when both pps and Hz are referred to in the same figure or context.

#### Data Analysis

##### AN Output

For faithfully coding ITD information, the peripheral stage needs to produce sufficient activity (quantified by spike rate) and phase locking. Phase locking refers to the synchronized firing of the simulated AN fibers to the pulse or to the envelope of the high-rate AM stimuli. The degree of phase locking can be quantified by the vector strength (Goldberg and Brown 1969). Each individual spike is represented as a unit vector with angle ${\varphi}_{k}$, corresponding to the spike time within the cycle. The vector strength *vs* is defined as

$$vs=\left|\frac{1}{N}{\sum }_{k=1}^{N}{e}^{(i{\varphi }_{k})}\right|,$$

with *N* being the total number of spikes and *k* indicating the *k*th spike. The vector strength becomes 1 if all spikes occur at a single phase of the stimulus waveform. Both the peristimulus time histogram (PSTH) and the period histogram were calculated to characterize the AN outputs. The model outputs to electrical pulse trains and acoustic stimuli were compared to the corresponding physiological observations (e.g., Dynes and Delgutte 1992; Javel and Shepherd 2000; Javel and Viemeister 2000; Joris and Yin 1992; Litvak et al. 2001).

##### EI Output

For illustrating the effect of different factors on the EI-model outputs, the simulated spike rates over a range of values were calculated (e.g., rate-ITD or rate-IPD or rate-level functions). To characterize the extent of ITD or IPD sensitivity from the rate-ITD or rate-IPD curves, typical metrics, such as the signal-to-total variance ratio (Chung et al. 2016; Hancock et al. 2013, 2010) cannot be directly employed, because the EI model is fully deterministic, i.e., the across-trial variance is expected to be unrealistically low. If one would assume Poisson noise, the square roots of the spike rates would have a constant variance, so that the sensitivity index d′ for discriminating between two ITDs would be proportional to the difference of the square roots of the two spike rates. This assumption is not necessarily met, especially under electric stimulation (Javel and Viemeister 2000). Nevertheless, to obtain a useful measure for the rate-ITD function modulation depth, orienting on the Poisson assumption is arguably more realistic than using the almost absent simulated variance: A best-case discrimination metric was chosen, contrasting between best and worst ITD, i.e., $\sqrt{{r}_{\mathrm{max}}}-\sqrt{{r}_{\mathrm{min}}}$, where *r*_max_ and *r*_min_ is the maximum and minimum spike rates, respectively. Furthermore, to quantify the similarity between an electric and an acoustic rate-IPD or rate ITD function, *R*^2^ values were obtained from various cross-correlations of the simulated spiking rates.

The model outputs in response to electrical pulse trains and acoustic stimuli were compared to the corresponding physiological observations. LSO neurons are sensitive to both ILD and the envelope ITD of AM tones (Joris and Yin 1995), and the acoustically stimulated EI-model outputs were compared to LSO data with corresponding stimuli (e.g., Joris 1996; Joris et al. 1998; Joris and Yin 1995). While there is no corresponding electrically stimulated LSO data as in the EI-model simulations, both the outputs of the electrical models of unmodulated low pulse-rate and modulated high pulse-rate stimuli were compared to IC recordings (e.g., Chung et al. 2016; Hancock et al. 2013; Smith and Delgutte 2007, 2008). However, it should be noted that neurons in the IC have ITD tuning that can resemble those of both the MSO and LSO, and can also exhibit a cross between the two types (McAlpine et al. 1998; Yin and Kuwada 1983).

## RESULTS

### AN Outputs

#### Acoustic Stimulation

To compare the acoustic model responses to Klug et al. (2020) and between different acoustic inputs, Fig. 2 shows the four types of acoustic stimuli with *f*_*c*_ = 4000 Hz (row 1), the inner hair cell (IHC) receptor potential (row 2), the spike raster plots (row 3), the PSTH, and the period histograms (row 4 and small insets) of AN fibers.

In general, the results of SAM tones are similar to those shown by Klug et al. (2020, Fig. 1, stimulation level of 20 dB SPL). Both PSTHs and the period histograms of the AN outputs show good phase locking to the 128 Hz modulation frequency for all types of stimuli. However, the synchronization, or the vector strength (*vs*), of filtered clicks and GE tones are higher than the other two in this example, which was expected when comparing the AC/DC ratio.

$$\mathrm{AC}/\mathrm{DC\;ratio}=\frac{\sqrt{\sum_{f\ne 0}{A}^{2}(f)}}{A(0)},$$

where *A*(*f*) and *A*(0) are the FFT amplitude of the rectified input stimuli at non-zero and 0 Hz, respectively (related to the *vs* of the output). Both filtered clicks and GE tones showed a higher AC/DC ratio than the other two stimuli.

##### Electrical Stimulation.

**Fig. 3:**
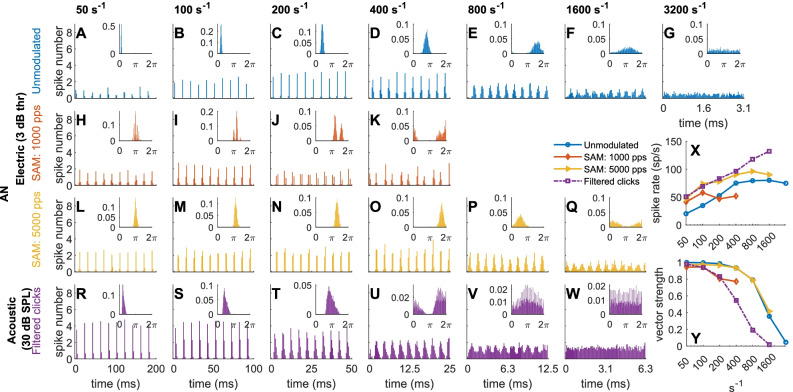
PSTHs and period histograms (small insets) of simulated AN responses to unmodulated pulse trains **A**–**G** at different pulse rates (in pps) and to amplitude-modulated stimuli at different *f*_*m*_ (in Hz), for 1000 pps **H**–**K** and 5000 pps **L**–**Q** at 3 dB thr. As a comparison, the outputs of acoustic stimulation (CF = 8000 Hz) at different pulse rates for the filtered clicks are plotted in the bottom panels (**R**–**W**, 30 dB SPL). The results shown in the figure were calculated by pooling all the responses from one example AN neuron with 20 repetitions for each condition. The ordinate of the PSTH is spikes per bin, with a bin size of 1 ms. The rate-frequency **X** and the *vs*-frequency **Y** functions are the spike rate and the vector strength over a range of pulse rates (for unmodulated pulse trains and filtered clicks, blue solid line with circles, and purple dash-dotted line with squares) or modulation frequencies (for SAM high-rate pulse trains at 1000 pps and 5000 pps carrier, orange and yellow solid lines with diamonds and triangles, respectively), with *x*-axis on a logarithmic scale. The unit s^−1^ refers to either pps or Hz

Figure 3 shows the output of one example electrically stimulated AN model neuron to unmodulated and to SAM high-rate electric pulse trains for various stimulation rates at 3 dB thr. For comparison, the outputs of one acoustically stimulated AN model neuron for the filtered clicks at a level of 30 dB SPL are shown in the bottom row.

In general, the vector strength (Fig. 3(Y)) gradually declined with increasing rate. At 200 and 400 Hz modulation frequency the phase locking to the modulation is worse with 1000 pps compared to 5000 pps, because these envelope cycles are only 5 and 2.5 times the inter-pulse interval at 1000 pps, respectively. A single pulse offset in the response (as seen in the small inset of Fig. 3(J)) results in a considerable difference with respect to the modulation cycle phase and thus to the lower vector strength.

The overall pattern of AN responses to unmodulated pulse trains (Fig. 3(A–G)) was consistent with physiological observations (Javel and Shepherd 2000; Javel and Viemeister 2000). When comparing SAM high-rate pulse trains (Fig. 3(H–K) or (L–Q)) with unmodulated pulse trains (Fig. 3(A–G)) of a rate corresponding to the modulation frequency of SAM stimuli, Fig. 3(Y) reveals that phase locking to unmodulated pulses was slightly higher in most cases (blue circles vs. orange diamonds and yellow triangles).

**Fig. 4:**
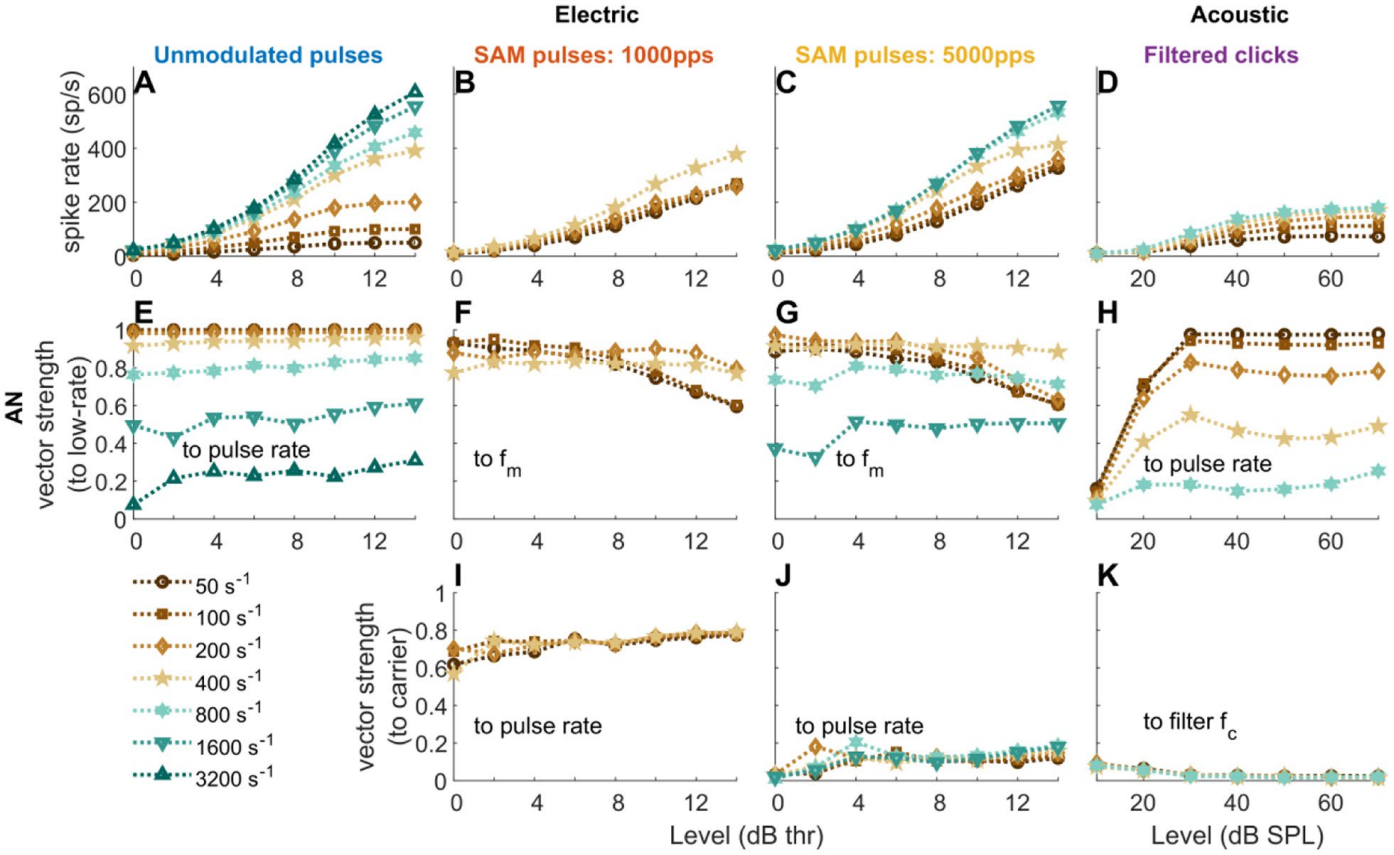
Average spiking rate-level functions **A**–**D** and *vs*-level functions **E**–**K** of electrical and acoustic stimuli (CF = 8000 Hz) at different pulse rates (20 repetitions): unmodulated pulse trains **A**, **E**; 1000 pps carrier SAM pulse trains **B**, **F**, and **I**; 5000 pps carrier SAM pulse trains **C**, **G**, and **J**; filtered acoustic click trains **D**, **H**, and **K**. For the unmodulated pulse trains, only the vector strength to the pulse rate is shown **E**. For the AM stimuli, vector strength is shown to both modulation frequency (2nd row) and to the carrier (3rd row). The unit s^−1^ refers to either pps or Hz

As expected at such low levels, the firing rate (Fig. 3(X), blue solid line with circles) is lower than the stimulation rate, indicating that not every stimulation cycle triggers a spike (Fredelake and Hohmann, 2012). For the 1000 pps carrier SAM pulses (Fig. 3(H–K)), phase locking exists to both the carrier and the amplitude modulation, causing the complex PSTH and period histogram shapes in Fig. 3. Phase locking to different components of the stimuli is shown in Fig. 4 in more detail as a function of stimulation level.

The stimulus level had a large effect on the neurons’ firing rate, and sometimes on its synchronization. For example, Joris and Yin (1992) showed that the behavior of AN fibers changes with the level of an acoustic AM signal. In our simulations, AN rate-level functions (Fig. 4(A–D)) showed the typical sigmoidal shape. It started to flatten out at 10 dB thr for unmodulated, low-rate pulse trains (Fig. 4(A), e.g., ≤ 100 pps), and near 12–14 dB thr for the other stimuli. The continuous increase for the SAM pulse trains (Fig. 4(B, C)) resulted from the increased firing during the modulation trough, which also caused the gradual decline of *f*_*m*_ synchrony with level at low modulation frequencies (Fig. 4(F, G), e.g., ≤ 100 Hz). At 12–14 dB thr, *f*_*m*_ synchrony was best at intermediate, and not at the lowest modulation frequencies (Fig. 4(F, G), e.g., between 200 and 400 Hz). The reason is that, at low *f*_*m*_ and high levels, the response rate exceeded the modulation frequency, i.e., multiple responses occurred within each modulation cycle. In line with (Dynes and Delgutte 1992; Miller et al. 2008), those simulated auditory nerve fibers, which have been allocated a low jitter, still showed some residual phase locking to electric stimulation up to 5000 pps (Fig. 4(J)), and strong phase locking to 1000-pps carrier pulses (Fig. 4(I)). Response synchrony to filtered clicks (Fig. 4(H)) was similarly level-independent as to unmodulated electric pulses (Fig. 4(E)), except near threshold, when spontaneous activity caused a lower synchrony, especially in the acoustic model (Fig. 4(H), < 30 dB SPL).

### Binaural Neuron Simulations

#### Acoustic Stimulation

**Fig. 5:**
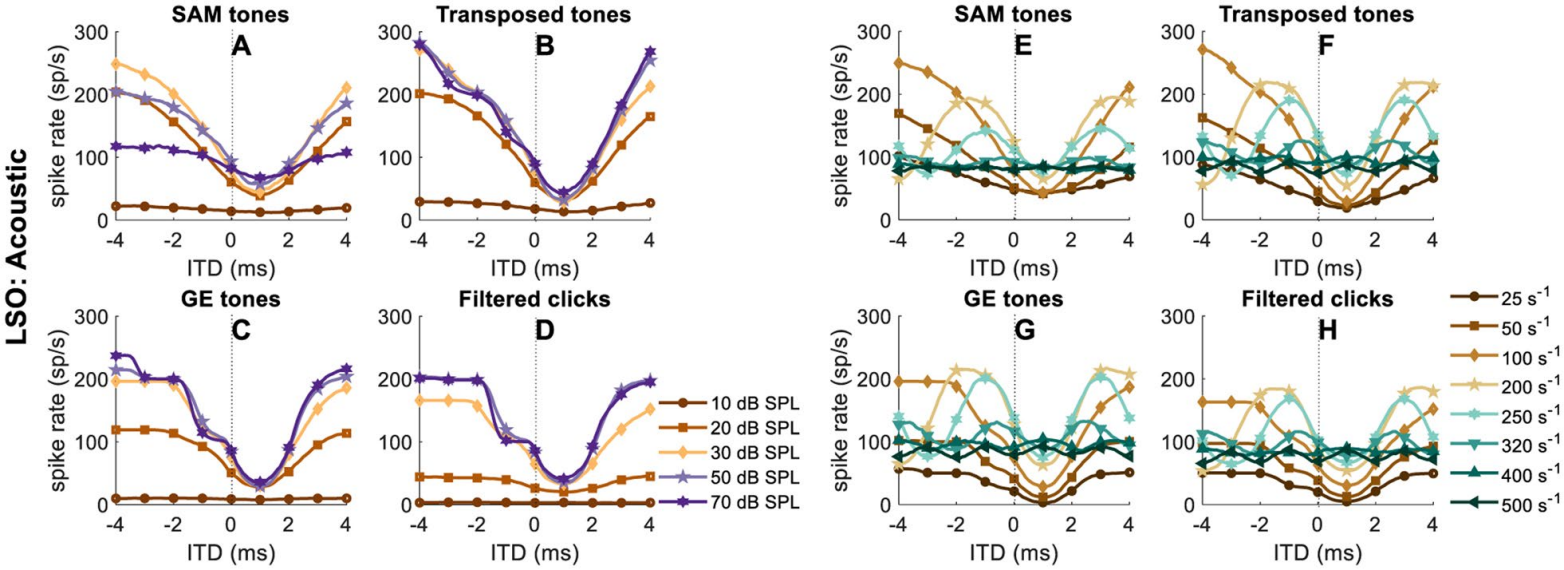
Rate-ITD functions **A**-**D** (10 repetitions, ITD step size of 0.1 ms, within ([-4,4]ms) of the EI-model neuron at different levels for different acoustic stimuli: *f*_*m*_ = 100 Hz and carrier frequency of 8000 Hz. Rate-ITD functions **E-H,** same format and neuron as in **A**-**D,** but now at a constant level of 30 dB SPL for different modulation frequencies. The unit s^−1^ refers to either pps or Hz

The average rate-ITD functions of the EI-model neuron at different levels for the four stimulus types are shown in Fig. 5(A–D). As in Klug et al. (2020, Fig. 3C, left hemisphere), the trough was not at zero ITD, because the inhibition lasted longer than the excitation. The minimum response was reached when the excitation was centered in the longer inhibition (Ashida et al. 2016; Klug et al. 2020). In general, the rate-ITD functions in Fig. 5(A–D) were sharper at higher levels for most stimuli; except for the SAM tones (Fig. 5(A)), the tuning curve became shallower with increasing levels above 30 dB SPL. The steps shown in the rate-ITD functions for the non-SAM stimuli at higher levels (Fig. 5(B–D)) were caused by the simplistic modeling of refractoriness at the EI stage. If the excitation was strong and preceded the inhibition by more than the refractory period (1.6 ms), a second response was generated within the same modulation cycle. This is why the plateau was at a spike rate that equals the modulation frequency. At the highest levels, a second plateau arose at an ITD of about 3.2 ms less than the worst ITD, at a rate twice the modulation frequency.

Figure 5(E–H) shows rate-ITD functions for different modulation frequencies (SAM and transposed tones) or different pulse rates (GE tones and filtered clicks) at a fixed level of 30 dB SPL. This is the level at which SAM tones evoked the highest peak spike rate in the simulated neuron (Fig. 5(A)) and does so in typical LSO neurons (e.g., FIG. 16B in Joris and Yin 1998). The rate dependence shown in Fig. 5(E–H) is also similar to experimental data (Joris and Yin 1998) and is primarily limited by the auditory-filter bandwidth

#### Electric Stimulation

##### The Effect of Input Level on the Rate-IPD Functions

**Fig. 6:**
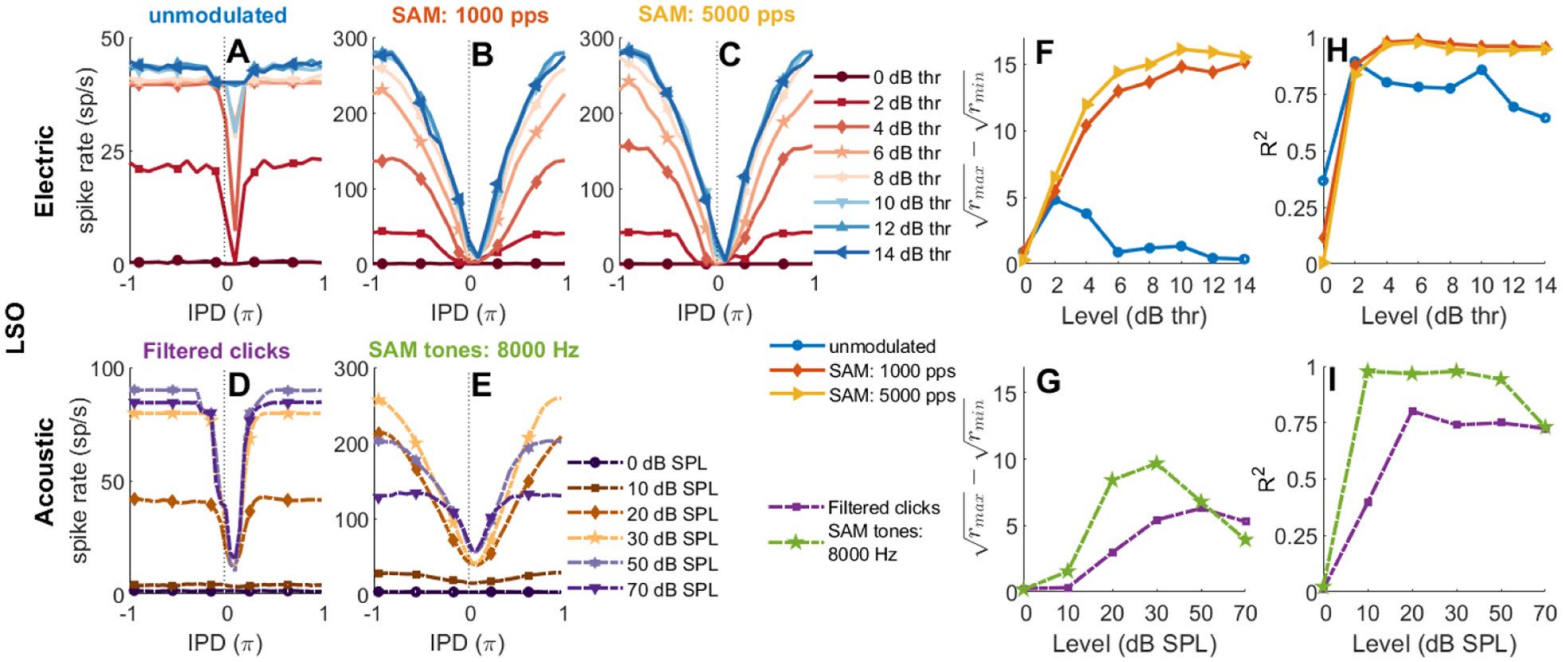
Average rate-IPD, $(\sqrt{r_{\text{max}}}-\sqrt{r_{\text{min}}})$-level, and the *R*^2^-level functions (20 repetitions, with IPD step size of 0.05$\pi$ within [$-\pi,\pi$]; positive IPDs represent contralateral-leading) obtained from the outputs of the electrically (solid lines) and acoustically stimulated (CF = 8000 Hz, dash-dotted lines) single EI-model neuron, respectively. The rate-IPD tuning curves of electrically stimulated EI-model include both unmodulated and modulated stimuli presented at different levels between 0 to 14 dB thr (with step size of 2 dB): 40-pps unmodulated pulse trains **A** and 40 Hz SAM high-rate pulse trains at a different carrier rate (**B**, 1000 pps; **C**, 5000 pps). The bottom panels are results from the acoustically stimulated EI-model at different levels ([0 10 20 30 50 70] dB SPL): 40-pps filtered clicks **D** and 40-Hz SAM tones **E**. The *y*-axes of the unmodulated pulse trains and the filtered clicks **A** and **D** are different to the rate-IPD plots of SAM stimuli **B**, **C**, and **E**. The $\sqrt{{r}_{\mathrm{max}}}-\sqrt{{r}_{\mathrm{min}}}$ functions are plotted in **F** and **G** for electrical and acoustic stimulation, respectively. **H** and **I** show *R*^2^ values between the rate-IPD function obtained with a reference stimulus and a rate-IPD function obtained with another probe stimulus of the respective opponent modality at different probe levels. Panel **H**, blue line with circles, shows the *R*^2^ from correlating the acoustic filtered click train at 20 dB SPL (reference stimulus, function from panel **D**, dark brown line with diamonds) with unmodulated electric click probes (all functions from panel **A)**. Panel **H** red line with diamonds and yellow line with triangle marks, the correlation of the 8000-Hz acoustic SAM tone at 20 dB SPL (brown line with diamonds from panel **E**) with the electric SAM pulse trains at 1000 pps from **B** and 5000 pps from **C**, respectively. Panel **I** purple line with squares shows the *R*^2^ from an unmodulated electric pulse train at 4 dB thr (reference, orange line with diamonds, panel **A**) and filtered click trains at different levels (probes, panel **D**, all curves). Panel **I** green pentagram symbols are the *R*^2^ values between the 5000 pps SAM pulse trains at 4 dB thr (reference, orange line with diamonds, panel **C)** and the 8000-Hz SAM tones at different levels (probes, panel **E**, all curves)

Figure 6 shows the rate-IPD (Fig. 6(A–E)), *R*^2^-level (Fig. 6(F, G)), and $(\sqrt{r_{\text{max}}}-\sqrt{r_{\text{min}}})$-level (Fig. 6(H, I)) functions of an electrically and an acoustically stimulated EI-model neurons at different levels. In order to characterize the similarity between two rate-IPD tuning curves, the *R*^2^ values were obtained, which is the square of the correlation coefficients between two rate-IPD tuning curves. For the rate-IPD tuning of unmodulated pulse trains at 40 pps, the effect of pulse level on the outputs of the EI-model neuron (Fig. 6(A)) was similar to experimental data from neurons with a trough- or step-type tuning curve (e.g., the neuron in the top-left panel of Figure 5 in Smith and Delgutte 2007). At intermediate levels (Fig. 6(A), e.g., ~ 2 dB thr), when the inhibition starts before and ends after the excitation, inhibition was able to stop almost all responses. At high levels (Fig. 6(A), e.g., > 10 dB thr), when all or almost all AN fibers entrain to the stimulation pulses, inhibition was not sufficient to stop the responses, because 20 excitatory minus 8 double-weighted inhibitory inputs resulted in a membrane potential of + 4, i.e., just enough to surpass the firing threshold of 3. At higher levels (Fig. 6(A), e.g., > 8 dB thr), some firing rates were higher than the pulse rate (40 pps) at larger IPD, because in some rare cases, one pulse triggered a second spike after the refractory period of 1.6 ms, i.e., triggered by the same effect as the plateaus in Fig. 5(B–D).

The differences between the rate-IPD functions of filtered clicks and SAM tones (Fig. 6(D, E), *f*_*m *_= 40 Hz) for different levels are more apparent than the differences between two stimulus types shown in Fig. 5(D, A, *f*_*m*_ = 125 Hz). For 40 Hz, the shape of the rate-IPD tuning curve of filtered clicks (Fig. 6(D)) is more similar to the unmodulated electrical pulse trains (Fig. 6(A)) than to the acoustic SAM tones (Fig. 6(E)), except that tuning remains pronounced for filtered clicks at high levels. The extent of similarity between the rate-IPD functions of filtered clicks and the unmodulated pulses, and between the SAM tones and SAM high-rate pulses, were further quantified in Fig. 6(H, I), respectively. In all cases, an acoustic level of 20 dB SPL and an electric level of 4 dB thr resulted in *R*^2^ values > 0.75 and generally remain high (> 0.7) for higher levels. Only for unmodulated electric pulse trains, a higher level leads to a reduction in shape similarity, because the modulation trough at IPD = 0 disappears.

#### Effect of Modulation Frequency or Pulse Rate on *IPD*_*ENV*_ Sensitivity

As noted in the sect. “INTRODUCTION”, a goal of this study was to investigate the rate limitation of ITD sensitivity with electrical stimulation. In the present model, it was expected to be limited by the length of the inhibitory window. As soon as the modulation or stimulation period is equal or shorter than this window, the inhibitory input is smeared out across several cycles and prohibits the normal emergence of a rate-ITD dependence in an EI neuron.

**Fig. 7:**
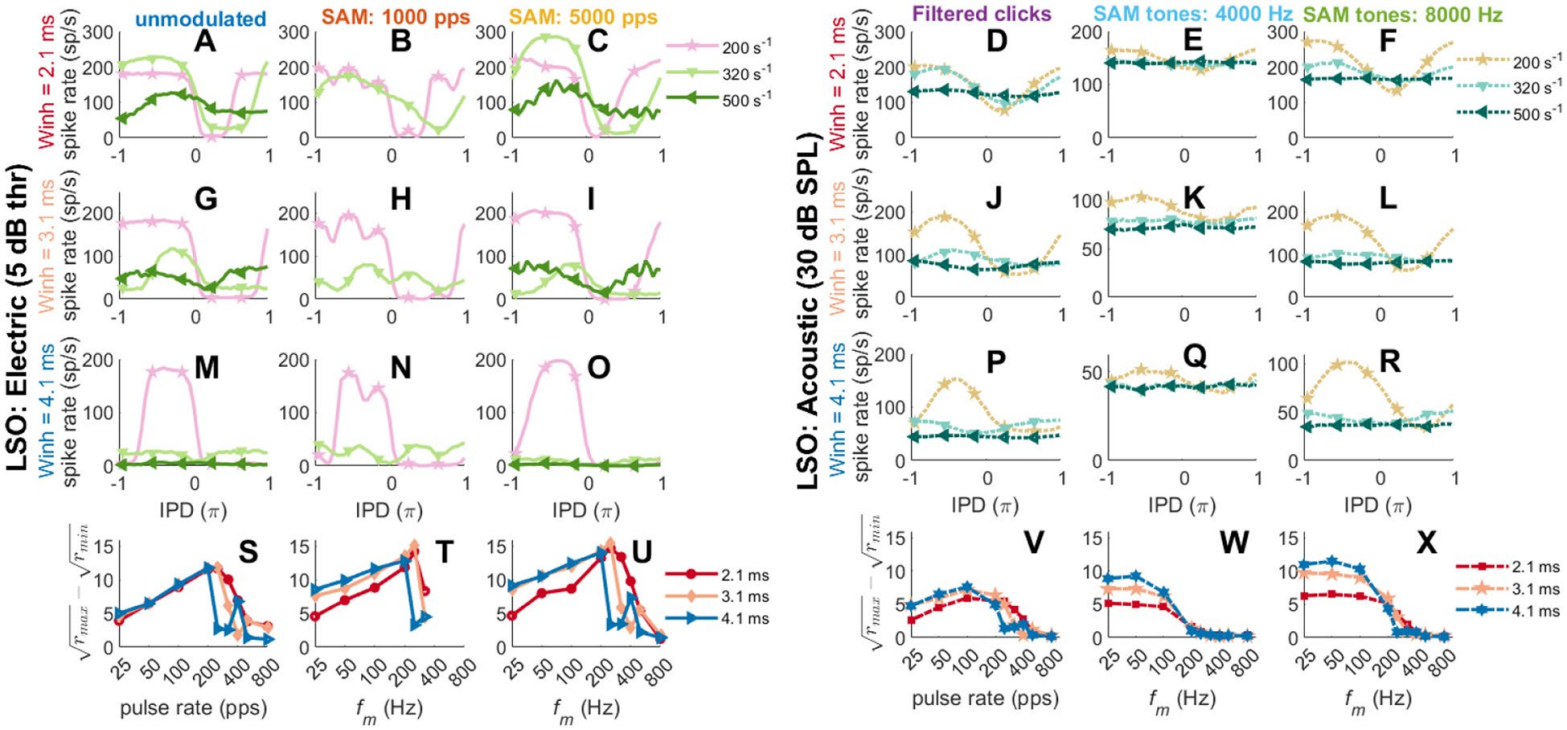
LSO rate-IPD tuning curves **A**–**R** and ($\sqrt{{r}_{\mathrm{max}}}-\sqrt{{r}_{\mathrm{min}}}$)-frequency **(**pulse rate or *f*_*m*_ ∈ [25 50 100 200 250 320 400 500 800] s^–1^) curves **S-X **(with IPD step size of 0.05$\pi$ within [$-\pi,\pi$]; positive ITDs represent contralateral-leading; three tested *W*_inh _values of [2.1 3.1 4.1] ms) for unmodulated **A**, **G**, **M**, **S**, SAM high-rate pulse trains at a carrier rate of 1000 pps **B**, **H**, **N**, **T** and 5000 pps **C**, **I**, **O**, **U**, filtered clicks **D**, **J**, **P**, **V**, SAM tones with carrier frequency of 4000 Hz **E**, **K**, **Q**, **W**, and 8000 Hz **F**, **L**, **R**, **X**, respectively. For the sake of clarity, only some rate-IPD curves of selected frequency between 200 and 500 s^−1^ are shown in **A–R**. Three different inhibition window lengths *W*_inh_ of 2.1 ms (red symbols), 3.1 ms (peach symbols), and 4.1 ms (blue symbols) are shown in each ($\sqrt{{r}_{\mathrm{max}}}-\sqrt{{r}_{\mathrm{min}}}$)-frequency subplot (electric, **S–U**; acoustic, **V**–**X**), where the *x*-axis is on a logarithmic scale. The results were calculated from 20 and 10 repetitions for electrical (at 5 dB thr) and acoustic stimulations (at 30 dB SPL). The unit s^−1^ refers to either pps or Hz

Until now, a fixed inhibitory window length (*W*_inh_) of 3.1 ms was used as in Klug et al. (2020). Figure 7 now shows the average rate-IPD curves for unmodulated (Fig. 7(A, G, M)), SAM high-rate pulse trains (Fig. 7(B, H, N; C, I, O)), filtered clicks (Fig. 7(D, J, P)), and SAM tones (Fig. 7(E, K, Q; F, L, R) for three different inhibitory window lengths (1st row, 2.1 ms; 2nd row 3.1 ms; 3rd row 4.1 ms) and the corresponding ($\sqrt{{r}_{\mathrm{max}}}-\sqrt{{r}_{\mathrm{min}}}$)-frequency curves in Fig. 7(S–X). In general, the decreasing of *W*_inh_ led to a higher *f*_*m*_ or pulse-rate limit. The default *W*_inh_ = 3.1ms corresponded to an upper rate limit of 323 s^−1^ as shown in Fig. 7(G–L). Up to this limit, IPD sensitivity does not critically depend on the spiking rate. Only at low stimulation levels was there a decline in the ($\sqrt{{r}_{\mathrm{max}}}-\sqrt{{r}_{\mathrm{min}}}$) value when lowering the pulse rate or the modulation frequency of electrical stimuli (Fig. 7(S–U)) and filtered clicks (Fig. 7(V)). The effect of *W*_inh_ was relatively smaller for the 4-kHz carrier acoustic SAM tones (Fig. 7(F, L, R, X)), due to the additional above-mentioned auditory-filter bandwidth limitation. Additionally, for the electric SAM stimuli at 1000 pps, envelope ITD sensitivity was compromised by the reduced envelope sampling accuracy above 200 Hz (Fig. 7(B, H, N, T)), especially if a short inhibitory window did not limit the sensitivity.

**Fig. 8:**
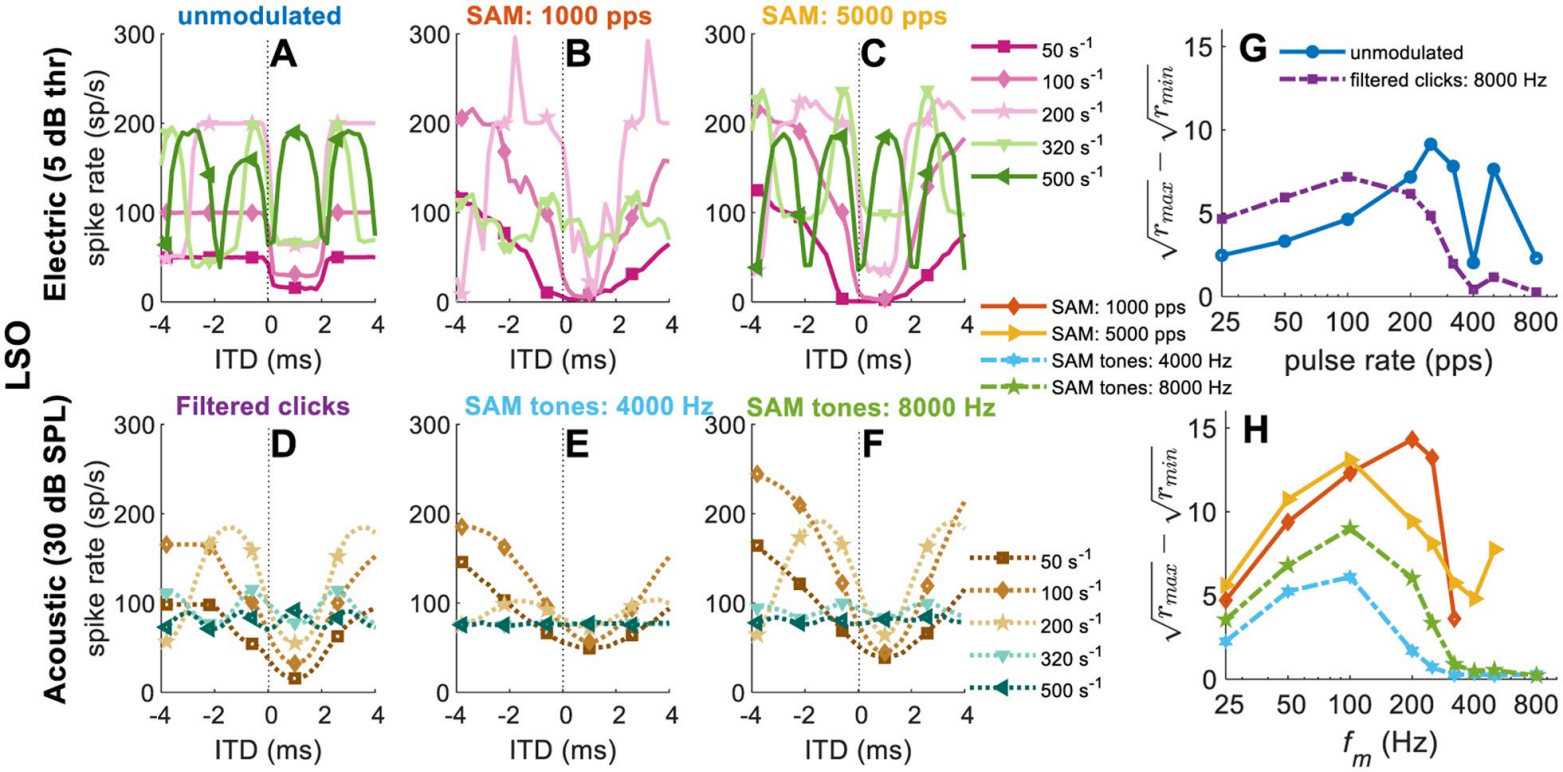
Rate-ITD tuning curves and ($\sqrt{{r}_{\mathrm{max}}}-\sqrt{{r}_{\mathrm{min}}}$) functions (with ITD step size of 0.2 ms within [-4,4] ms; positive ITDs represent contralateral-leading, pulse rate or *f*_*m *_∈ [25 50 100 200 250 320 400 500 800] s^–1^) for electrical and acoustic stimulation: top panels are rate-ITD functions for unmodulated **A** and SAM high-rate pulses (**B**, 1000 pps; **C**, 5000 pps) at level 5 dB thr; bottom panels are the rate-ITD functions for the filtered clicks **D** and the SAM tones (**E**, 4000 Hz; **F**, 8000 Hz) at 30 dB SPL. The ($\sqrt{{r}_{\mathrm{max}}}-\sqrt{{r}_{\mathrm{min}}}$) values for different pulse rates are shown in **G** (unmodulated pulse trains, blue solid line with circles; filtered clicks, purple dash-dotted line with squares). The ($\sqrt{{r}_{\mathrm{max}}}-\sqrt{{r}_{\mathrm{min}}}$) values for different modulation frequency are shown in **H** (SAM high-rate pulse trains, 1000 pps, orange solid line with diamonds; SAM high-rate pulse trains, 5000 pps, yellow solid line with triangles; SAM tones, 4000 Hz, blue dash-dotted line with hexagrams; SAM tones, 8000 Hz, green dash-dotted line with pentagrams). For the sake of clarity, only the rate-ITD curves of selected frequencies between 50 and 500 s^−1^ are shown in **A**–**F**. The results were calculated from 20 repetitions for acoustic and electric EI-model outputs, respectively. The *x*-axis of **G** and **H** is pulse rate (pps) and modulation frequency (Hz) on a logarithmic scale, respectively. The unit s^−1^ refers to either pps or Hz

So far, the complete IPD cycle was tested, which corresponds to an increasing ITD range with increasing pulse rate or modulation frequency. However, a fixed range of ITDs has commonly been tested experimentally (e.g., Chung et al. 2016; Joris and Yin 1995; Smith and Delgutte 2008). Figure 8 shows average rate-ITD functions and ($\sqrt{{r}_{\mathrm{max}}}-\sqrt{{r}_{\mathrm{min}}}$)-functions for a commonly tested ITD range of ± 4 ms.

In general, the cyclic behavior of the SAM stimuli was better captured by an IPD metric (Fig. 7), but the more transient filtered clicks and unmodulated pulses revealed the influence of the model’s time constants better on an ITD metric (Fig. 8). For the unmodulated pulse trains, the model simulations were generally consistent with published responses from some IC neurons (e.g., Figure 2D in Chung et al. 2016). In addition, the lower sustained spiking rate in the electric response than in the acoustic response at high pulse rates (e.g., Fig. 7(A, G, M) vs. (D, J, P), IPD = 0, 500 pps) was in line with (Su et al. 2021). As expected, the EI model cannot reproduce peak-type rate-ITD functions, as also commonly observed in some studies (Smith and Delgutte 2007, 2008).

For the SAM high-rate pulse trains (Fig. 7(H, I) and Fig. 8(B, C)), the EI-model outputs were generally consistent with Smith and Delgutte (2008): (1) The shapes of the rate-IPD tuning curves were fairly stable for lower *f*_*m*_ (e.g., below 100 Hz), although the peak firing rates varied substantially, consistent with Smith and Delgutte (2008, e.g., their FIG. 4A, from 40 to 160 Hz). Consequently, the rate-ITD functions get sharper with increasing modulation frequency. (2) The rate-IPD functions of 1000 pps SAM pulse trains were modulated by both the modulation frequency and carrier rate, especially for *f*_*m *_≥ 200 Hz. The reason for such carrier-rate modulated oscillations can be understood when comparing the interaural pulse amplitude difference for each pulse pair, introduced by applying the ITD_ENV_. This can also be an explanation for the restoration of sustained responses to high-rate pulse trains by low-frequency AM, as suggested in Smith and Delgutte (2008, e.g., their FIG. 12). (3) At low modulation frequencies (*f*_*m*_ ≤ 100 Hz), the EI outputs are nearly identical for both carrier frequencies, in line with Smith and Delgutte (2008, e.g., their FIG. 4D and FIG. 5A,B). Regarding the upper *f*_*m*_ limit, Smith and Delgutte (2008, e.g., their FIG. 5B) showed ITD_ENV_ sensitivity up to the highest tested *f*_*m*_ of 160 Hz. Previous studies suggested that there is a modulation frequency limit near 200–300 Hz (Snyder et al. 1995, 2000), similar to the rate limit with constant-amplitude pulse trains. Although using a higher carrier rate increased the upper limit of *f*_*m*_ that could be delivered in SAM high-rate pulse trains, due to the inhibitory window length 3.1 ms, the cutoff modulation frequency for all stimuli was close to 320 Hz.

For the acoustically stimulated EI-model outputs, the *f*_*m*_-dependence of the rate-ITD functions was very similar to those recorded in the LSO (e.g., Figure 11 in Joris and Yin 1995, ~ 200/300 Hz for 5/12.4 kHz CF). In addition to the limitation from the inhibitory window, ITD sensitivity is *f*_*m*_-limited by the width of the auditory filter (see also Fig. 7(W and X)).

## GENERAL DISCUSSION

In this study, the response of an EI-model neuron with acoustic or electric stimulation was compared to LSO and IC recordings. In general, the EI-model is able to reproduce most characteristics of acoustically stimulated LSO neurons (Joris 1996; Joris and Yin 1998) and of step-type or trough-type IC neurons in the case of both electrical (e.g., Chung et al. 2016; Smith and Delgutte 2007, 2008) and acoustic stimulations (e.g., Dietz et al. 2016; Greenberg et al. 2017; Griffin et al. 2005). That said, the majority of reported rate-ITD functions to unmodulated electrical pulse trains do not match in shape those reported here. In part, this is simply due to ours showing a larger ITD range beyond the ± 2 ms that is typically applied experimentally. A clear mismatch remains for the narrowly tuned, peak-type responses, prominent in Smith and Delgutte (2007; 2008) and Vollmer (2018). Those response patterns can be expected to originate from fast excitatory-excitatory (EE) type interaction, as commonly associated with MSO processing, and can be simulated with existing models (Chung et al. 2015; Colburn et al. 2009). These models, in turn, cannot simulate the EI-type patterns.

The peak-type neurons are also ITD sensitive at high pulse rates, such as 1000 pps (e.g., Smith and Delgutte), as a consequence of the short excitatory postsynaptic potentials (EPSPs) of MSO inputs, and are markedly different to our simulations or to typical step-type or trough-type neurons (Chung et al., 2016). On the other hand, the simulated pulse-rate limitation of ITD sensitivity near 320 pps is markedly similar to the limit observed in step-type or trough-type IC neurons under electrical- (Chung et al. 2016) or acoustic stimulation (Greenberg et al. 2017; Griffin et al. 2005). This limit is also comparable to the ITD sensitivity limit of human bilateral CI users (e.g., Ihlefeld et al. 2015) and to the envelope ITD sensitivity limit of normal-hearing humans (Bernstein and Trahiotis 2014). As previously hypothesized (Dietz 2016; Kelvasa and Dietz 2015), all of this hints at a dominant role of EI interaction in binaural processing under electrical stimulation, in line with the larger negative binaural interaction component in bilaterally implanted CI users (Brown et al. 2019; Gordon et al. 2012; He et al. 2012; Hu and Dietz 2015). Future studies need to examine whether such a single pathway model, which can also simulate ILD sensitivity within the very same model neuron (Klug et al. 2020), is fully sufficient to model binaural perception of an average bilateral CI user. Currently, this appears to be possible. Individual rate-limit differences in the 250–500 s^−1^ range can be simulated with different inhibition time constants or, more biophysically, with different synaptic properties (Brughera et al. 2021; Dietz et al. 2016). Only for the occasional sensitivity well above 500 pps (e.g., van Hoesel et al. 2009) is an additional MSO model branch expected to be essential. However, no satisfactory answer can be given as to why EE processing may not be accessible to electrical stimulation in many bilaterally implanted humans. The idea is only loosely based on descriptions of MSO and LSO circuits, hinting at different degrees of robustness: the ITD tuning of fast, highly leaky, MSO neurons have already developed in the juvenile system by optimizing myelination patterns (Stange-Marten et al. 2017) and an accurately tailored expression of inhibitory inputs (Beiderbeck et al. 2018; Brand et al. 2002; Grothe and Pecka 2014). We speculate that any form of input changes may corrupt this type of ITD sensitivity. On the other hand, the simple EI mechanism can be expected to be robust against input variations (Joris and Yin 1995; Tsai et al. 2010) and is also the reason for CI users’ fair sensitivity to ILD (Hancock et al. 2010). While the EI-model output does critically depend on the number of input fibers, these differences can be compensated for, by adapting other model parameters such as the response threshold (Klug et al. 2020).

Two aspects that are not in line with physiology, or potentially subject to controversy, are the absence of a cochlear nucleus and some other processing stages, as well as the rectangular-shaped 3.1-ms inhibition window. This nominal length is much larger than commonly reported from LSO neurons (Sanes 1990). In part, this is due to the different definition. The equivalent rectangular duration is usually larger by a factor of 2–3 than time constants from an integration window function. For the present simplistic EI model, the difference between the inhibition time constant and the excitation time constant is also relevant and determines the duration of troughs in some of the rate-ITD functions to be just over 2 ms (Fig. 8(A)).

In any case, we do not want to make anatomical claims about where the EI interaction happens. The LSO is one possible location, but *de novo* EI interaction at the level of the IC is also an option. In the absence of physiological recordings to electric stimulation from the brainstem, this question cannot be answered. The rectangular window shape, however, is a simplification that can be problematic for the highly phase-locked electrical input. For a more detailed study of the course of the ITD-sensitivity decline with increasing electrical pulse rate, a more realistic window shape would be required.

In addition to the ITD rate limit, the effect of input level on the rate-ITD or rate-IPD functions was also very similar to experimental data of electrically stimulated IC neurons (e.g., Fig. 5, trough- or step-type, Smith and Delgutte 2007) or acoustic stimulated LSO neurons (e.g., FIG. 16B in Joris and Yin 1998). In general, there are increased response rates and sharpening ITD tuning curves with increasing level before saturation effects reduce tuning at high levels. Some discrepancy remains to the generally increasing behavioral ITD sensitivity with increasing level (e.g., Egger et al. 2016), but a similar discrepancy is known from acoustic hearing (e.g., Dietz et al. 2013). In order to better relate the results to psychophysical abilities of bilateral CI users, the model has to be combined with suitable back-ends for discrimination or lateralization tasks. As with acoustic stimulation (Klug et al. 2020), the model is expected to be generally able to account for both ILD- and ITD-based lateralizations with the same EI-model units. For example, an ongoing follow-up project is to connect the current EI-outputs with the most simplistic rate-difference decoding back-end used in (Klug et al., 2020) to predict different lateralization and localization data reported in previous bilateral CI experiments (e.g., Egger et al. 2016; Ihlefeld et al. 2015; Kan et al. 2016; Laback et al. 2015, 2004; Stakhovskaya and Goupell 2017). It may be prudent, or even necessary, to model a range of EI neurons across the tonotopic array. Such a population is expected to cause a more gradual change of lateralization with increasing ITD (e.g., Baumgärtel et al. 2017), despite the step-wise rate-ITD tuning functions observed both experimentally and in simulated neurons. It is also expected to account for the abovementioned discrepancy in level dependence.

The EI-model stage can also be embedded into model frameworks that include CI signal processing, or at least multi-electrode stimulation (e.g., Kelvasa and Dietz 2015; Stakhovskaya and Goupell 2017; Todd et al. 2016). Simulating the consequences of, e.g., coding strategy or interaural mismatches in insertion depth, neural health, or fitting parameters on localization is a possible application. Compared to the Hodgkin-Huxley-based MSO or LSO models of previous studies (e.g., Chung et al. 2015; Kelvasa and Dietz 2015), it offers the advantage of computational speed and more intuitive parametrization at the cost of physiological detail, such as the rectangular window shape.
